# Macroscopic and microscopic perspectives for adoption of technologies in the USA

**DOI:** 10.1371/journal.pone.0242676

**Published:** 2020-12-03

**Authors:** Alexsandro M. Carvalho, Sebastián Gonçalves, Janaína Ruffoni, José Roberto Iglesias

**Affiliations:** 1 Escola de Gestão e Negócios, Programa de Pós-Graduação em Ciências Contábeis, UNISINOS, Porto Alegre, RS, Brazil; 2 Instituto de Física, Universidade Federal do Rio Grande do Sul, Porto Alegre, RS, Brazil; 3 Escola de Gestão e Negócios, Programa de Pós-Graduação em Economia, UNISINOS, Porto Alegre, RS, Brazil; 4 Instituto Nacional de Ciência e Tecnologia de Sistemas Complexos, CBPF, Rio de Janeiro, RJ, Brazil; Consejo Nacional de Investigaciones Cientificas y Tecnicas, ARGENTINA

## Abstract

Adoption of a new technology depends on many factors. Marketing, advertising, social interactions, and personal convictions are relevant features when deciding to adopt, or not, a new technology. Thus, it is very important to determine the relative weight of these factors when introducing a new technology. Here we discuss an agent based model to investigate the behavior of agents exposed to advertising and social contacts. Agents may follow the social pressure, or maybe contrarians, acting against the majority, to decide if they adopt or not a new technology. First, we solve analytically the model that relies on the above quoted factors. Then, we compare the theoretical results with empirical data concerning the adoption of innovations by American households during the 20th century. The analysis of the diffusion dynamics process is done either for the whole period, or by periods based on the so-called technical-economic paradigms, according to Freeman and Perez. Three different periods are considered: before 1920, from 1920 to 1970, and after 1970. We study the evolution of the model parameters for each technical-economic period. Finally, by adjusting the key parameters we are able to collapse all the data into a universal curve that describes all the adoption processes.

## 1 Introduction

The economic development of a society depends on the generation and application of new technologies (See, for example, Ref. [1]). New products and methods are essential to the dynamics of markets and to the growth of the economy in a macro-scale level. New technologies originates in the accumulation of capacities and resources to be used in the production of goods and services [2]. From an economic point of view, an innovation only assumes full relevance when it spreads on all its potential users. Without diffusion, innovation has no economic impact. It is the process of diffusion that allows to transform the innovation, originally an event secluded in time and space, into a phenomenon with relevant meaning in industries and services [3]. The diffusion of an innovation is the process in which economic agents (individuals or firms) adopt a new technology, or replace an old process by a new one. The adoption process is the result of several individual decisions of the utility of innovation [4]. But even if the decisions are isolated, they are influenced by advertising and/or by the interaction with other agents: the social contacts [5].

Early studies on the evolution and constraints of the diffusion of innovations processes appeared in the literature in the post-war period. They were developed from epidemiological models used to describe the dynamics of mass epidemic contagions in the population [6–8]. Given the assumed immutability of the products during the diffusion process and the predictable behavior of the adopters, *epidemiological models* were criticized during the ‘70s and ‘80s. To overcome these limitations and at the same time to incorporate new aspects in the dynamics of adoption, like learning processes derived from the adoption of new technologies, Probit’s class models emerged in the ‘70s [9–11]. Concomitant with Probit’s class models, the so-called *mixed influence models* [2, 5] appear in the literature. It is worth noticing that, while Probit and epidemiological models consider adoption from the firm perspective, mixed influence models also take into account the consumer point of view into the adoption dynamics.

Probably, the first and most known mixed influence model is the Bass model [5], which considers innovative and imitative behavior as the key factors for the timing of purchases (adoption) of new consumer products. The model considers that part of the process of adoption of innovation comes from learning or imitation of other adopters (social pressure), while spontaneous decisions are responsible for early adoption. Following Bass model, evolutionary models emerged thereafter to explore the role of learning in the diffusion of a new technology. In recent years, some authors have investigated the influence of the structure of contacts between adopters in the diffusion of innovation [12, 13]. Even more recently, using a model of interacting agents, Gonáalves et al. [14] have shown that the role of agents that resist to changes, *contrarians*, is also an important factor, as they may influence the adoption of new products, but also determine the timing at which this new product is adopted.

Another relevant aspect of innovation diffusion refers to the historical moment in which this process occurs. This means considering relevant historical events related to the structural aspects of the technological change. In this sense, Freeman and Perez [15] proposed a long cycles analysis of economic growth, centrally considering the characteristics of technological change. The theoretical basis of this discussion is Schumpeter [1] (1942) and Kondratieff [16] (1935); the latter author introduced the idea of long waves (50 years) to understand the dynamics of capitalism. Freeman and Perez [15] also introduced the concept of *technical-economic paradigm*, which is conceptualized as a combination of innovations that increase investment opportunities and profit. This paradigm is the result of the process of selecting a set of innovative combinations that permeate the entire economy and influence it. This concept is also important because it reveals that *one major characteristic of the diffusion patterns of a new techno-economic paradigm is its spread from the initial industries or areas of application to a much wider range of industries and services and the economy as a whole* (quoting Ref. [15], p.48). Freeman and Perez [15] mention that this is a “meta-paradigm”, emphasizing its potential relevance in certain explanations of technological change in society.

Freeman and Louçã [17] reinforce this understanding, emphasizing that the process of evolution of economies occurs through successive waves of structural change that emerge from technological revolutions. And the long waves of capitalist development are explained by the emergence and diffusion of new technologies. In terms of periodization, Freeman and Perez [15] distinguish five paradigms: 1) mechanization (1770-1840); 2) steam engines and railways (1840-1890); 3) electrical and heavy engineering (1890-1920); 4) Fordism (1920-1970); and Information and Communication Technologies (ICT) (1970-present period). An important feature of this last ICT stage is the ability to rapidly develop and introduce new products in the market. The innovation generation and diffusion speed are, in general, higher in this period relative to others. Rovere [18] (p. 293) points out that *as the process of globalization accelerates, capital and technology flows move faster*. It is interesting to note also that the US, the country we focus in this article, is one of the main protagonists in the last two paradigms mentioned: Fordism and ICT.

Therefore, the analysis of the process of diffusion by periods is justified, related to the paradigms identified before. The precise identification of each period is not obvious, because changes are gradual over time. Considering this, and the available data that we present in the next section, we assume here that it is important to capture the changes from the initial period of paradigm shifts, considering: 1) before 1920; 2) from 1920 to 1970, and 3) from 1970 until today.

In this contribution, we present an analytical solution for the dynamics of an agent model of innovation adoption. The model includes the influence of advertising, individual resistance to change, contrarians, and social influence.

The contrarians are agents acting against the majority, as described by Galam [19]. Different types of behaviors, like people disappointed or frustrated with some results or consequences of new technologies, could be called contrarians too. We have considered the effect of the “repented” agents in Ref. [20]; however, a full analysis of frustrated agents, as well as of the people who follow fashion, is outside the scope of this contribution and we will consider it elsewhere.

We adjust the parameters of the model using data for the US during the XXth century and analyze the change of the parameters along the economic waves. The paper is organize as follows: in the next section we present the data of diffusion of innovations in the US during the 20th century along with the agent model numerical and analytical results for each of the innovations. In Section III we present the results of fitting the US data with the model, in section IV we present an universal curve that fits all the data and we discuss the change of the parameters in time according to the periods of time corresponding to changes in paradigms. Finally, in section V we discuss the results and conclude.

## 2 Material and methods

### 2.1 Data

**Fig 1:**
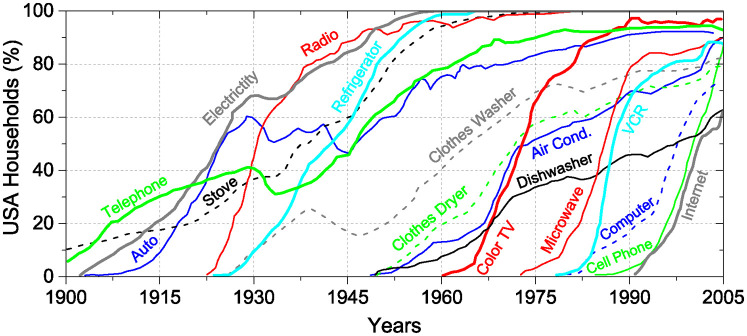
Adoption of technologies by households in the United States. Data obtained by digitization of the original plots by Felton published in the New York Times [21].

In 2008 Felton published in *The New York Times* a graph representing the dynamics of adoption of new technologies by US households during the 20th century [21]. The adoption curves of sixteen technologies, from electricity and automobile to cell phone and the Internet are represented in that plot, which we reproduce here (the data for the figure were obtained by digitizing the original one published in the NYT) in Fig 1. We can see in that figure that eventually all technologies attain a *plateau* or saturation. However, Felton points out that more recent technologies arrive at saturation faster than older ones. For example, electricity or the stove took a longer time to arrive full adoption, than color TV or the Internet. Nevertheless, it is worth mentioning that some technologies, like B&W TV (not mentioned in Felton’s article), have been adopted in the 1950s, in the United States, as fast as the Internet or the cell phone nowadays.

### 2.2 Model

**Fig 2:**
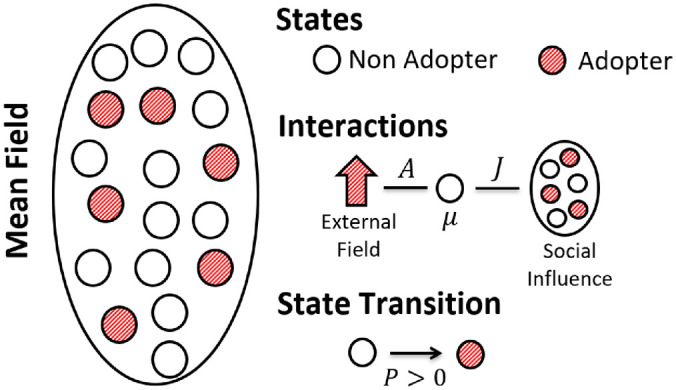
Schematic representation of the model: Each agent is represented by a circle. Full (red) circles are adopters and empty circles, non-adopters. The ellipse represents the close system with full mixed (mean field) interactions. Agents have individual resistance to adopt, *μ*_*i*_, and each of them feels the influence of everyone else through the interaction parameter, *J*. They are all subjected to the external field *A*, which includes both the external pressure to adopt (advertising) and the attractiveness of the good, either because of its utility or its price. Agents change their state when the payoff of the innovation, given by Eqs (1) or (2), are positive.

One of the aims of the present contribution is to describe the curves of adoption of technology based on a simple model inspired in Bass ideas [5]. But, differently form his phenomenological approach, the model we use starts from the microscopic dynamical description of agents exposed to a novelty, as schematically represented in Fig 2. We especially address the speed of adoption and the final percentage of adopters. To do so, we follow Ref. [14] and consider a system of *N* agents with full mixing interaction, that is, each agent interacts with all other (see Fig 2). Agents may be in one of two possible states: *adopter* or *non-adopter* of the innovation, being *N*_*a*_ the number of adopters and *N*−*N*_*a*_, that of the non-adopters. Those numbers are not static; according to Rogers [8], there are a given number of precursors or early adopters, followed by those that may adopt at a later time, mainly because of social pressure or herding effect [5]. So, *N*_*a*_ evolves in time.

We assume each agent has a resistance to adopt, *μ*_*i*_, representing both its reluctance to change technology and the amount of investment needed to acquire the new product. This resistance to adopt is an idiosyncratic feature, usually different for each agent, then we consider (as in ref [14]) that the values of *μ*_*i*_ are uniformly distributed within the interval [0, 1), so the average value is $\bar{\mu }=0.5$. Second, each agent is assumed to be under the influence of an external field *A* (Fig 2), that represents the pressure to adopt the new technology. This parameter condenses the advantages of the innovation and also the advertising of the developers and/or manufacturers of the new product. Third, each agent is subjected to the influence of the society trough a parameter *J* > 0. The social influence term can be positive (promoting more and faster adoption) or negative (restraining the adoption process) depending on the kind of agent. We call *mimetics* the agents that imitate the decisions of the others, while agents that act opposing to the herd, we name *contrarians* [14]. In both cases, the social interaction strengh is proportional to the fraction of adopters, *n* = *N*_*a*_/*N*, but while *mimetics* agents experience a positive term, *Jn*, *contrarians* are negative influenced; therefore, the social effect for the latters has a minus sign, *i.e.* the term is −*Jn*. The number of contrarians is *N*_*c*_, so the fraction of contrarians is *f*_*c*_ = *N*_*c*_/*N* and the fraction of mimetics, 1−*f*_*c*_.

To determine if an agent *i* adopts or not, we evaluate its payoff using the following equations [14], where one assumes all terms have the same units:

$$\begin{array}{c} P_{i}^{M}=A-\mu_{i}+Jn \end{array}$$ (1)

if the *i*−agent is mimetic or

$$\begin{array}{c} P_{i}^{C}=A-\mu_{i}-Jn \end{array}$$ (2)

if the *i*−agent is a contrarian. For simplicity, we assume that *A* and *J* have the same value for all agents, while the individual behaviors are embedded in the distribution of *μ*_*i*_. Besides, *A* has to be positive, otherwise nobody will ever adopt.

#### 2.2.1 Numerical solution

**Fig 3:**
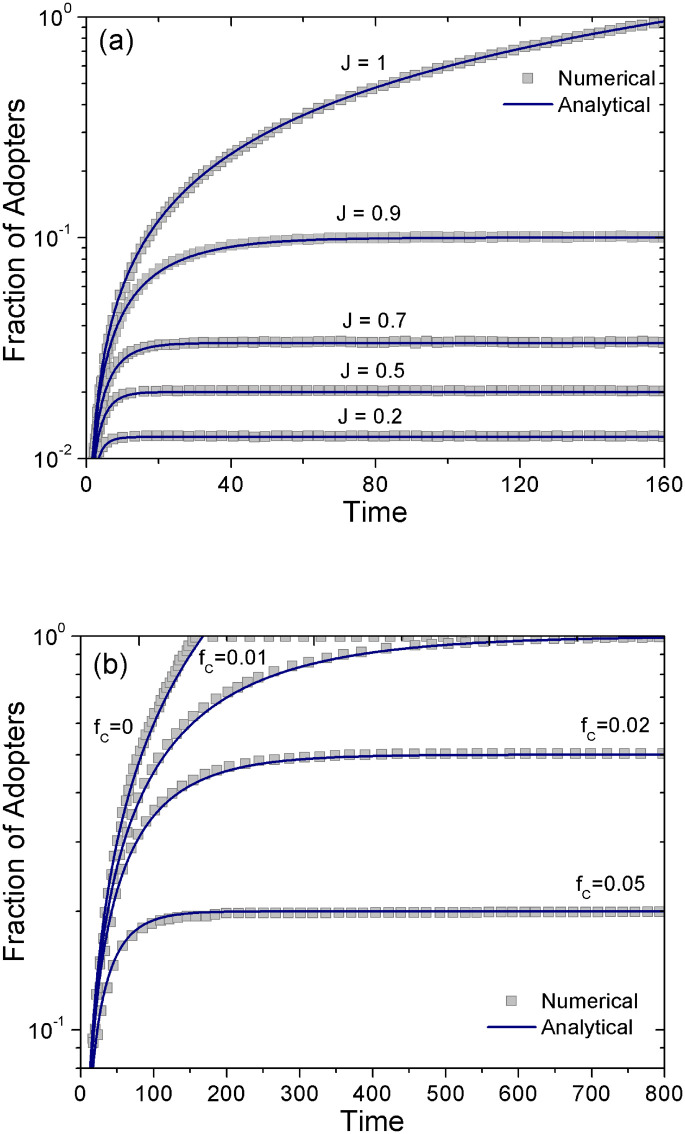
Fraction of adopters as a function of time. (a) Considering different values of the coupling constant *J* for no contrarians (*f*_*c*_ = 0). (b) Fraction of adopters for *J* = 1 and different values of *f*_*c*_. Continuous curves correspond to the analytic solution and squares to the numerical MC solution. In all cases *A* = 10^−2^ and *N* = 10^7^.

We present first a very brief description of the numerical solutions obtained in Ref. [14]. The numerical simulations were performed using the Monte Carlo (hereafter MC) method [22]. We consider an initial configuration of *N* non-adopters agents, each one characterized by an idiosyncratic resistance to adopt *μ*_*i*_ obtained from an uniform distribution in the interval [0, 1], with average value $\bar{\mu }=0.5$. The values of *μ*_*i*_, *A* (the external pressure to adopt), and *J* (the strength of the social interaction) are constant during the dynamics. Each time-step we choose at random and sequentially, *N* agents, and we evaluate their payoffs. Non-adopters agents with *P*_*i*_ > 0 will adopt, remaining in the same state otherwise. The updating of the *N* agents constitute one MC-step after which the number of adopters *n* is updated. Fig 3 shows numerical results for some cases along with the analytical solution we describe in the next subsection.

In Ref. [14], we have studied the effect of limiting social influence to a subset of each agent, rather than the entire population. However, such consideration, in addition to preventing analytical treatment, would introduce new parameters and in this work we prefer to restrict them to a minimum.

The probability of an agent being chosen randomly in a population of size *N* is 1/*N*. Consequently, 1 − 1/*N* indicates the probability of not being selected. Since the choices are independent, the probability of an agent not being selected at all in a MC-step is (1 − 1/*N*)^*N*^. Conversely, the probability of an agent to be selected at least once is $\lambda =1-{\left(1-\frac{1}{N}\right)}^{N}$. Then, for large values of *N* (we use *N* = 10^7^) we have *λ* ≈ 1 − *e*^−1^. The process terminates when the number of adopters no longer changes in time. Ref. [14] has already discussed the numerical results for this model in detail; therefore, in the next subsection, we limit ourselves to presenting the analytical results.

#### 2.2.2 Analytic solution

In this section we focus on the analytical evaluation of *N*_*a*_(*t*), the number of adopters at time *t* ∈ [0, ∞), using standard methods of stochastic calculus and considering full mixed interactions. *N*_*a*_(*t*) is given by a stochastic process where a non-adopter can change to an adopter state with probability functions *p*_*j*_(*t*) = *P*{*N*_*a*_(*t*) = *j*} where *j* is the number of adopters at time t. In particular, we consider that the transition probability at a given time depends exclusively on the previous time step (Markov process). So, the probability of a transition from state *N*_*a*_(*t*) = *j* to state *N*_*a*_(*t*+ Δ*t*) = *i*, *j* → *i*, in the time Δ*t*, is

$$\begin{array}{c} p_{ij}\left(\Delta t\right)=P\left\{N_{a}\left(t+\Delta t\right)|N_{a}\left(t\right)\right\}\;, \end{array}$$ (3)

where *p*_*ij*_ is the standard conditional probability. Given that agent *i* becomes adopter whenever its payoff (Eqs 1 and 2) is positive, which happens if

$$\begin{array}{ccc} & A+Jn>\mu_{i} & \left(mimetic\right) \end{array}$$ (4)

$$\begin{array}{ccc} & A-Jn>\mu_{i} & (contrarian)\;, \end{array}$$ (5)

and because the *μ*_*i*_ are uniformly distributed en [0.1), the above conditions happen with the following probabilities:

$$\begin{array}{c} p_{i}^{M}=\min\left(1,A+Jn\right),\;\;\;p_{i}^{C}=\max\left(0,A-Jn\right) \end{array}$$ (6)

respectively for mimetics and contrarians. Then, considering that *f*_*c*_ is the fraction of contrarians, the average probability of an agent in the population becoming a new adopter, at each time step, is

$$\begin{array}{c} \frac{\left(1-f_{c}\right)Np_{i}^{M}+f_{c}Np_{i}^{C}-N_{a}}{N}=\left(1-f_{c}\right)p_{i}^{M}+f_{c}p_{i}^{C}-n, \end{array}$$ (7)

which, using Eqs 6 and 7, reduces to

$$\begin{array}{c} A-(1-J^{\prime })n, \end{array}$$ (8)

where *J*′ = (1 − 2*f*_*c*_)*J*. Considering that in the time interval Δ*t* these interactions occur with a frequency *ω*. So, the transition rate, *i.e.* the transition probability *j* → *j*+ 1 per unit time, is

$$\begin{array}{c} p_{ij}\Delta t=\omega [A-(1-J^{\prime })n]\Delta t=\Omega \left(j\right)\Delta t. \end{array}$$ (9)

Complementary, the probability of not changing state, *j* → *j*, is 1 − Ω(*j*)Δ*t*. Thus, the transition probability is given by

$$\begin{array}{c} p_{ij}\left(\Delta t\right)=\{\begin{array}{c} \Omega (j)\Delta t+o(\Delta t),\;i=j+1\\ 1-\Omega (j)\Delta t+o(\Delta t),\;i=j \end{array} \end{array}$$ (10)

The term *o*(Δ*t*) is an infinitesimal included to keep valid the expression for small values of Δ*t* (lim_*t* → ∞_ *o*(Δ*t*)/Δ*t* → 0). Assuming *P*(*N*_*a*_(0) = *j*_0_) = 1 and $p_{jj_{0}}\left(t\right)=p_{j}\left(t\right)$, we have

$$\begin{array}{ccc} p_{j}\left(t+\Delta t\right)= & & p_{j-1}\left(t\right)\Omega \left(j-1\right)\Delta t+\\ & + & p_{j}\left(t\right)(1-\Omega (j)\Delta t)+o\left(\Delta t\right). \end{array}$$ (11)

Subtracting *p*_*j*_(*t*) and dividing by Δ*t* (Δ*t* → 0), we obtain

$$\begin{array}{c} \frac{dp_{j}}{dt}=p_{j-1}\Omega \left(j-1\right)-p_{j}\Omega \left(j\right) \end{array}$$ (12)

for *j* = 1, 2, …, *N* and *dp*_0_/*dt* = 0. Now, we rewrite Eq 12 using the finite differences method where the unit step is Δ*j*,

$$\begin{array}{ccc} \frac{dp_{j}}{dt} & = & -\frac{p_{j+1}\Omega \left(j+1\right)-p_{j-1}\Omega \left(j-1\right)}{2\Delta j}+\\ & + & \frac{p_{j+1}\Omega \left(j+1\right)+p_{j-1}\Omega \left(j-1\right)-2p_{j}\Omega \left(j\right)}{2{\left(\Delta j\right)}^{2}} \end{array}$$ (13)

or, for *p*_*j*_(*t*) = *p*(*j*, *t*) and Δ*j* → 0

$$\begin{array}{c} \frac{\partial p(j,t)}{\partial t}=-\frac{\partial }{\partial j}[\Omega (j)p(j,t)]+\frac{1}{2}\frac{\partial^{2}}{\partial j^{2}}[\Omega (j)p(j,t)]. \end{array}$$ (14)

Note that this equation is a forward Kolmogorov differential equation. An explicit solution is not possible because of the nonlinearities. However, it can be shown that *p*(*j*, *t*) is the probability distribution of solutions to the Itô stochastic differential equation [23]

$$\begin{array}{c} \left\{ \begin{array}{c} dN_{a}\left(t\right)=\Omega \left(N_{a}\left(t\right)\right)dt+\sqrt{\Omega (N_{a}\left(t\right))}dW\left(t\right)\\ N_{a}\left(0\right)=N_{a}^{0} \end{array}\right. \end{array}$$ (15)

where *dW*(*t*) is a Wiener Process.

In order to determine the behavior of the average number of adopters 〈*N*_*a*_(*t*)〉, we take the mean values in both sides of Eq 15, obtaining

$$\begin{array}{ccc} \frac{d\langle N_{a}\left(t\right)\rangle }{dt} & = & \left\langle \Omega \left(N_{a}\left(t\right)\right)\right\rangle +\left\langle \sqrt{\Omega (N_{a}\left(t\right))}\frac{dW(t)}{dt}\right\rangle . \end{array}$$ (16)

For large values of *N*, the second term of the right side goes to zero and using Eq 9 one obtains:

$$\begin{array}{c} \frac{d\langle n_{a}\left(t\right)\rangle }{dt}=\omega [(A-\left(1-J^{\prime })\langle n_{a}(t)\right\rangle ], \end{array}$$ (17)

where 〈*n*_*a*_(*t*)〉 = 〈*N*_*a*_(*t*)〉/*N* is the fraction of adopters, subject to initial condition 〈*n*_*a*_(*t*_0_)〉 = *n*_0_. Eq 17 is a linear differential equation of first order that can be rewritten as

$$\begin{array}{c} \frac{d\langle n_{a}\left(t\right)\rangle }{dt}+\omega (1-J^{\prime })\langle n_{a}(t)\rangle =\omega A, \end{array}$$ (18)

with a general exponential solution plus an asymptotic particular solution for *t* → ∞ (which corresponds to $\frac{d\langle n_{a}\left(t\right)\rangle }{dt}=0$) equal to

$$\begin{array}{c} \langle n_{a}\left(t\right)\rangle =n_{\infty }-(n_{\infty }-n_{0})e^{-\omega (1-J^{\prime })(t-t_{0})}, \end{array}$$ (19)

where

$$\begin{array}{c} \langle n_{a}(\infty )\rangle \equiv n_{\infty }=\frac{A}{1-J^{\prime }} \end{array}$$ (20)

Note that the growth rate is *ω*(1 − *J*′). In Fig 3 we can see that the general analytical expression Eq 19 agrees with the numerical solutions of the model.

### 2.3 Data adjustment

We now turn to the use of the analytical solution (Eq 19) to explain the available data on different products adoption. In other words, to fit the analytical solution of the model to the empirical data [21], by adjusting the parameters *ω*, *J*′ and *A*. The three parameters are bounded: 0 < *ω* ≤ 1, 0 < *J*′ < 1, and 0 < *A* ≤ 1 − *J*′. The last inequality comes from the fact that the final fraction of adopters must lie in the interval 0 ≤ *n*(∞)≤1. The other inequalities arise from the definition of the parameters: *ω* is the frequency of interaction and *J*′ the effective social coupling. Remark that the condition *A* ≤ 1 − *J*′ and *J*′ ≥ 0 result from 0 ≤ *J*′ ≤ 1. As a consequence of this last inequality the fraction of contrarians and the interaction parameter must be *f*_*c*_ ≤ 0.5 —generally, it does not make much sense to have more than 50% of the population against a novelty—, and 0 ≤ *J* ≤ 1/(1 − 2*f*_*c*_), respectively.

Within the above mentioned constrains, the determination of the parameters is a problem of convex optimization with constraints [24], that we solve making use of a method of random optimization [25].

To fit the curves we choose the origin of the temporal series as the year of start of each technology [21] and we represent the fraction of adopters as a function of time in years. To check the quality of the adjustment we consider both the dynamical relative residual error (that we call Δ) and the average relative residual error (called 〈Δ 〉). The relative residues quantify how much the model fit deviates from the empirical curve year to year, and it is defined as:

$$\begin{array}{c} \Delta (t)=\frac{\left|n_{E}(t)-n_{A}(t)\right|}{\left|n_{E}(t)\right|} \end{array}$$ (21)

where *n*_*E*_(*t*) and *n*_*A*_(*t*) refer respectively to the empirical and analytic fraction of adopters at time *t*. The second measure is the global average of the residues over all observations in time given by:

$$\begin{array}{c} \left\langle \Delta \right\rangle =\frac{1}{t_{f}+1}\sum_{t=0}^{t_{f}}\Delta \left(t\right) \end{array}$$ (22)

where *t*_*f*_ is the final time of the empirical temporal series.

## 3 Adjust and study of the model parameters

### 3.1 Fitting the data

**Fig 4:**
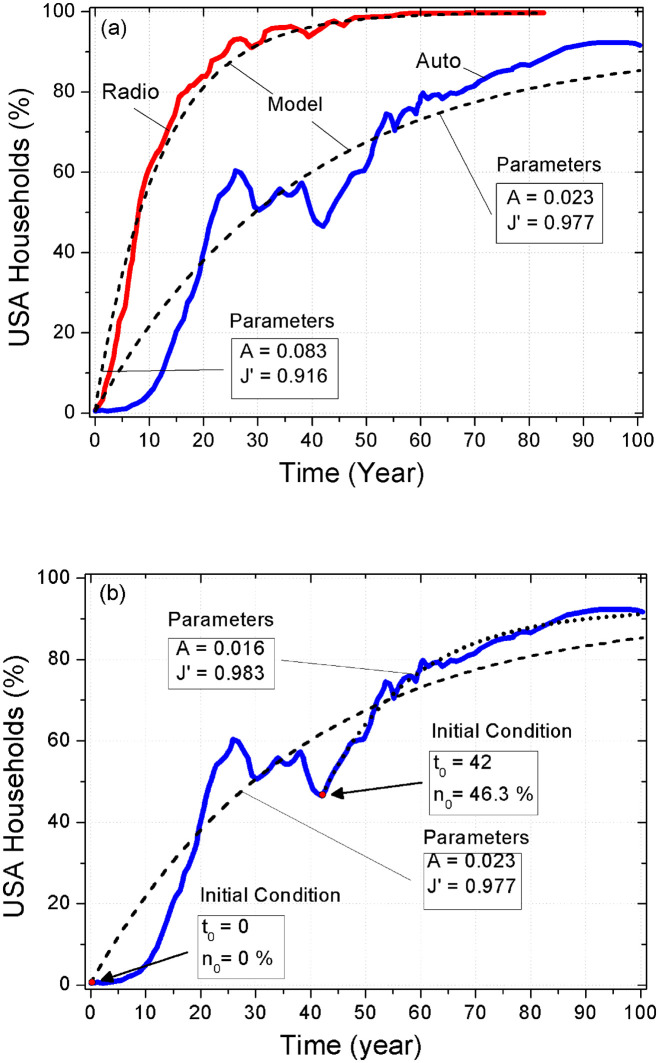
Dynamics of adoption of new technologies by American households, according to the data of Felton [21]. Continuous lines correspond to Felton’s data for two selected innovations, radio and automobile. Dashed lines correspond to the best fit using analytic solution Eq 19 with initial conditions *n*(*t*_0_) = *n*_0_. (a) Adoption of radio and automobile are represented from the inception of the innovations, *i.e.* with *n*(0) = 0. The adjustment is much better for radio than for car, because of the big fluctuations of car production during World War II. (b) Automobile adoption using two different starting points, *n*(0) = 0 (dashed line, with year 0 corresponding to calendar year 1903) and considering a new beginning after the World War II, *n*(42) = 0.463 (dotted line, with year 42 corresponding to calendar year 1945). The fit after the World War II is much better than for the full time-lapse. *ω* ≃ 1 for both technologies.

Fig 4a shows the best fit using Eq 19 for the dynamics of adoption of the radio and the automobile, starting from the dawn of the new technology, i.e. *n*(0) = 0. One can verify that the model follows the tendency in both temporal series. However the precision of the fit depends both on time and on the technology considered. On the other hand, Fig 4b represents the fit just for the case of the automobile but with two different starting points. The curve from 0 to 100 is the same as in Fig 4a, that is, the time starts at the beginning of the innovation (year 1903). Still adoption of automobiles suffered from shortages of raw materials, particularly iron and rubber, during World War II, and this is evident because the fraction of adopters exhibit a minimum in the year of 1945. So, we made a second fit placing the origin of the time evolution at the moment of the minimum, and this second fit presents a much smaller deviation from the original data than the previous one. The values of the parameters are described in the figure.

**Fig 5:**
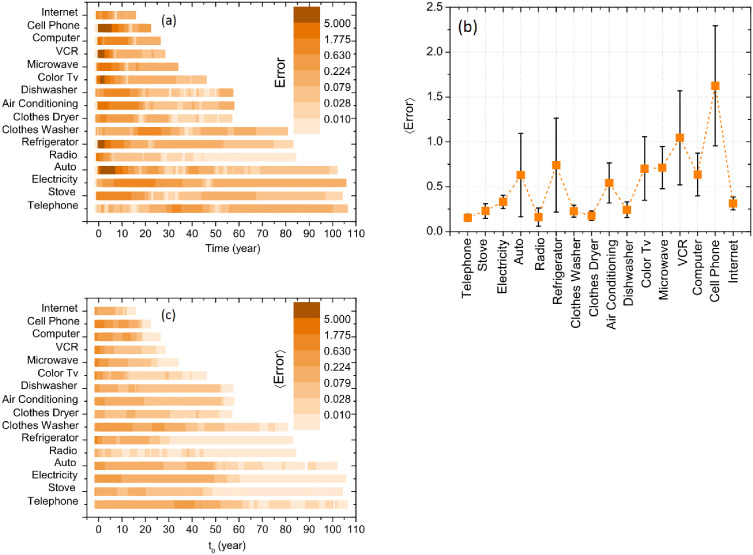
Quality of the data fits for each of the technologies analyzed, measured by the relative error between the empirical curves [21] and the analytical solution (Eq 19, after fitting. Errors are calculated considering the onset of the innovation, *i.e.* *n*(0) = 0: (A) Dynamic relative error (Eq 21), (B) Average relative error (Eq 22). Vertical bars represent the standard deviation of the results and the dotted line is a guide for the eyes. (C) Average relative errors for different initial conditions *n*(*t*_0_) = *n*_0_. Initial conditions are different for each technology.

To measure the goodness of the fits, we present in Fig 5a the positive relative differences between the fit values and the actual ones, along the time, for all the innovations. We call these differences as dynamical residues (Δ, Eq 21); the less they are, the best are the fittings. Clearly, the residues are bigger during the first years of adoption, as expected, with the exception of the telephone and the cloth washing machine. As an example, for the automobile the residue is bigger than 1.775 for the first ten years of adoption while in the last decade the relative residue is lower than 0.079.

To compare the quality of the fit for the different technologies, we plot the time averaged residues 〈Δ 〉 on Fig 5b. One can verify that the fit is better for the radio (minimum value of 〈Δ 〉) than for the cell phone (maximum value of 〈Δ 〉). We arbitrarily separate the quality of the fits in four sets:

〈Δ 〉<0.25 Low residue: telephone, stove, radio, clothes washing machine, clothes dryer, dishwasher.
0.25 ≤ 〈Δ 〉<0.5 Low to intermediate: electricity and internet.
0.5 ≤ 〈Δ 〉<0.75 Intermediate to high: automobile, refrigerator, air conditioning, color TV, microwave oven and computer.
〈Δ 〉≥0.75 High: VCR and cell phone.

All the results described so far are associated with fits of data from the onset of the innovation. However, with a different choice of the initial conditions, the quality of the fits improves considerably. We have already show on Fig 4b a fit of the data of adoption of automobiles considering as starting point *n*(42) = 46.3%, the minimum adoption condition at the end of WWII. It is evident that the fit is better for this starting time and the evaluation of the residues confirms this impression: the average residue is 〈Δ (*t*_0_ = 0)〉 ≈ 0.63 for *t*_0_ = 0 and 〈Δ (*t*_0_ = 42)〉 ≈ 0.17 for *t*_0_ = 1942.

Following this method we present on Fig 5c the average relative residues for different initial times for different technologies. We can observe that the average residue 〈Δ 〉 diminishes when varying the initial time *t*_0_. In general, the value of the relative residual is bigger when considering the full series, with the exception of the telephone.

### 3.2 Macroscopic perspective: Diffusion speed and final number of adopters

The speed of adoption of an innovation is directly related to the rate of adoption, that is, the number of new adopters per unit time. We measure the instantaneous speed by considering the marginal adoption, *V* = *dn*_*a*_/*dt*. This marginal adoption may be obtained from the model by direct differentiation of the analytic expression given by Eq 19. To obtain the speed of adoption from the empirical data, we have to differentiate numerically by the method of finite differences. As the speed is a function of time, to compare different technologies, we consider the average value of adoption rate along all the adoption time, *i.e.* 〈*V*〉 = 〈*dn*_*a*_/*dt*〉.

**Fig 6:**
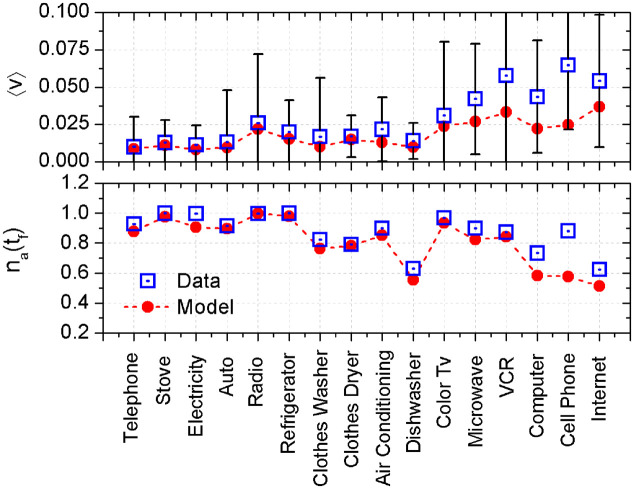
Speed of diffusion, 〈*V*〉 (top panel) and final fraction of adopters, *n*_*a*_(*t*_*f*_) (low panel) for the different technologies. All values have been determined from the onset of the new technology. Squares correspond to the empirical data and circles to the theoretical model. Bars represent the standard deviation. Dotted lines are guides for the eyes.

We represent in the top panel of Fig 6 the speed of diffusion obtained from the data for different technologies (squares). We remark that the cell phone exhibits the higher speed of adoption, roughly 6% a year or 〈*V*〉 = 0.06*year*^−1^ while the conventional telephone has the lower speed, 1% a year: 〈*V*〉 = 0.01*year*^−1^. Comparing speeds we can divide innovation in two groups, the first group from conventional telephone to dishwasher exhibit a low speed of adoption, while more recent technologies from color TV to the Internet exhibit higher speed of adoption. In average, innovations from the second group have a speed of adoption three times higher than those of the first group. In the same figure, circles represent the results of the model. The tendencies are the same for empirical and theoretical results, but the model results are less accurate for recent innovations, probably because the time series are shorter for new technologies.

Finally, in the low panel of Fig 6 we represent the final fraction of adopters, *n*_*a*_(*t*_*f*_), for each technology, (squares represent empirical data and circles the model). With a few exceptions all innovations are adopted by almost the full population. Some differences arise from the time an innovation is present. For example, electricity started in the 19th century, while the Internet and the cell phone are relatively recent technologies. Old technologies are in the final state of full adoption, while recent ones are still growing. However, we must remark that by 2014, the number of cell phones *per capita* in the USA was 1.03 [26], clearly attaining full adoption.

In any case, one can verify that the results of the model (circles) are in good agreement with the data (squares). So we can use the parameters obtained in fitting the curves to estimate if and when a recent technologies will arrive to full adoption.

### 3.3 Microscopic perspective: The behavior and influence of the parameters

**Fig 7:**
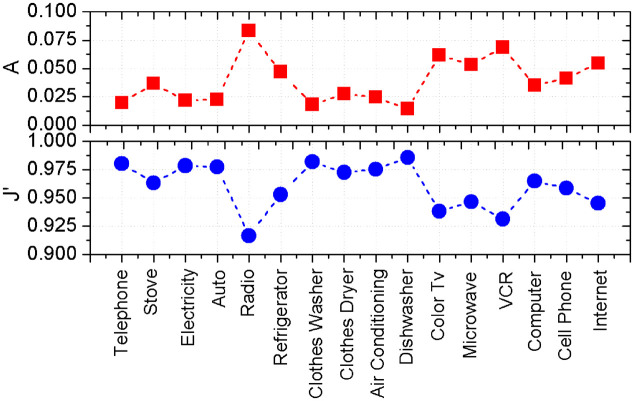
Parameters of the model *vs* technology. The values of *A* (top panel) and *J*′ (low panel) are the result of the best fit of the empirical data with the analytical solution of the model. *ω* ≃ 1 for all the technologies.

It is implicit in the works of Rogers [8] and Bass [5] that all adoption curves have a qualitative similar behavior. In this subsection we introduce a renormalization of the parameters in order to put in evidence the universal behavior of adoption. We verify that the sets of parameters collapse in a linear relation, then we propose a transformation that make all adoption curves to be represent in a universal one. We start by showing in Fig 7 the values of the advertising, *A* (top panel), the effective coupling parameter that describes social interactions, *J*′ (middle panel) for the different technologies represented in the data [21]. The values of the parameters have been obtained by adjustment using Eq 19. We emphasize that all curves have been fitted starting at *t*_0_ = 0, the dawn of the innovation.

First, we inspect the parameter that measures the pressure by the sellers/producers of the technology, *A*. The extreme values of this parameter are the ones of the radio and the dish-washer, *i.e* *A*_*max*_ = 0.083 (radio) and *A*_*min*_ = 0.014 (dish-washer). It is also striking that relatively recent technologies like color TV or the Internet exhibit a parameter *A* as high as that of the radio, and with approximately twice the value of other technologies.

The effective coupling parameter, *J*′, exhibits a behavior complementary with that of *A*, but the values are confined between 0.9 and 1.0, for example the maximum value of *J*′ is $J_{max}^{\prime }=0.9857$ (dis-washer) while the minimum one is $J_{min}^{\prime }=0.9167$ for the radio. This complementary behavior is not surprising. Being done that the final fraction of adopters is of the order of one, from Eq 20 one obtains:

$$\begin{array}{c} A\approx 1-J^{\prime }. \end{array}$$ (23)

**Fig 8:**
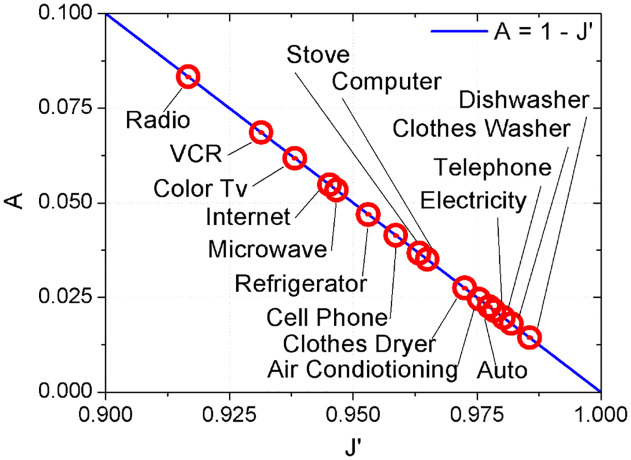
Best-fit parameters of the model represented in the *A* *vs* *J*′ plane. Each point (symbol ⊙) represents the pair of values of the best fit of the empirical data with the analytical solution of the model. The solid line (blue) corresponds to the *A* = 1 − *J*′ relation. *ω* ≃ 1 for all the technologies.

The above relation has been plotted in Fig 8 where we have represented each technology by the pair of values of the parameters *A* and *J*′ that best fit the adoption curves. The fact that the points lie on the the line implies that the value of *ω* can be taken as

$$\begin{array}{c} \omega \approx 1 \end{array}$$ (24)

for all technologies, implying that all agents interact with the external influence and with the other agents at the same time-step. If Eq 23 is valid, then, the parametes *A* and *J*′ should be in a straight line for all technologies; that is precisely what it is shown in Fig 8. Besides, that implies that all adoption curves mainly depend on a single parameter. As we have the freedom of choosing *A* or *J*′ as the independent parameter, if one consider *A* as the independent one, substituting Eqs 23, 24 in Eq 19, and assuming the initial condition *n*(0) = 0, the universal relation

$$\begin{array}{c} \langle n_{a}\left(t\right)\rangle \approx 1-e^{-At} \end{array}$$ (25)

is obtained. The speed of diffusion is then given by:

$$\begin{array}{c} \langle V\rangle =\left\langle \frac{d\langle n_{a}\rangle }{dt}\right\rangle \approx A\langle e^{-At}\rangle . \end{array}$$ (26)

As a consequence 〈*V*〉 ∝ *A*. Confirming this result, the Pearson coefficient *R* between the speed of diffusion (Fig 6) and the advertising *A* is *R* = 0.7685, suggesting a strong correlation.

### 3.4 Technological-economic paradigms

Even if Fig 8 indicates that almost all technologies lies on a single relation between the pressure to adopt, *A*, and the social interaction modulated by the fraction of contrarians, *J*′, it is not clear if innovations corresponding to similar time lapses have similar values of the these parameters. One expect that different phases of technology development should correspond to different forms of adoption. We have observed that the profile of adoption curves is similar for most innovations, but it is relevant to compare the parameters that adjust each one of them.

In Table 1 we present a classification of the different technological innovations here studied accordingly with the year of introduction of this innovation. The first group corresponds to innovations that appeared before 1920: telephone, electricity, stove and automobile. Most of them appeared at the beginning of the 20th century. The second group correspond to innovations appearing between 1920 and 1970: radio, refrigerator, clothes washer, dishwasher, air conditioning, clothes dryer and color TV. Here the birth of the innovations is spread over an interval of more than forty years. Finally, the last group correspond to recent innovations that appeared after 1970: microwave oven, VCR, personal computer, cell phone and the Internet. For each technology we have calculated the values of *A* and *J*′ over the period of time considered.

**Table 1:** Dynamic of the parameters by period.

Dynamic of the Parameters by Period								
Groups	Technology	Beginning of Series [Year]	Period					
			0 < *t* < 20		20 < *t* < 70		*t* > 70	
			*A*	*J* ′	*A*	*J* ′	*A*	*J* ′
I	Telephone	1900	0.068	0.889	0.019	0.981	0.643	0.316
	Electricity	1900	0.021	0.979	0.035	0.965	0.422	0.576
	Stove	1902	0.020	0.980	0.065	0.935	0.466	0.534
	Auto	1903	0.012	0.988	0.571	0.287	0.321	0.667
Parameters (Average) Beginning of Diffusion			〈 *A* 〉	〈 *J* ′ 〉				
			0.030	0.940				
II	Radio	1922			0.083	0.917	0.354	0.645
	Refrigerator	1923			0.045	0.954	0.966	0.029
	Clothes Washer	1925			0.015	0.985	0.814	0.000
	Dishwasher	1949			0.112	0.889	0.015	0.985
	Air Conditioning	1949			0.013	0.986	0.468	0.531
	Clothes Dry	1949			0.021	0.978	0.328	0.584
	Color TV	1960			0.029	0.971	0.444	0.547
Parameters (Average) Beginning of Diffusion					〈 *A* 〉	〈 *J* ′ 〉		
					0.045	0.954		
III	Microwave	1973					0.053	0.947
	VCR	1978					0.068	0.931
	Computer	1980					0.035	0.964
	Cellphone	1986					0.041	0.956
	Internet	1990					0.054	0.945
Parameters (Average) Beginning of Diffusion							〈 *A* 〉	〈 *J* ′ 〉
							0.050	0.949

Values of *A*, *J*′ and 〈*V*〉 for each technology, separated according to the time of introduction of the new technology. The symbols 〈*A*〉 and 〈*J*′〉 refer to the mean values of the parameters for each group. Period I, before 1920, period II, 1920-1970, and period III, after 1970. Values in red correspond to period I, green to period II, and blue to period III.

On the bottom of the table the mean value of the parameters is represented for the first group, called *I*, the second, *II* and the last one, *III*. One can observe a correlation among the values of the parameters and the technical-economic paradigm period. The average value of the pressure to adopt, *A*, decreases as times goes by, and the value of the social interaction increases. Also, innovations are adopted faster in the recent paradigm than in the previous ones, in agreement with the hypothesis of the NYT article [21]. This is also in conformity with the observed effect of social networks that are determinant in many choices nowadays [14].

Calculations with different values for the beginning and end of economic waves have also been performed. The numerical results are slightly different but exhibiting the same tendency, so they seem to confirm that technological innovations are strongly correlated with technical-economic paradigms.

## 4 Conclusions

We have shown that with a simple analytic calculation on a model of social interaction [14] inspired on the original work of Bass [5], is possible to adjust the curves of adoption of new technologies with just two parameters: the pressure to adopt, *A*, and the effect of social pressure, *J*′. The effect of “contrarians”, agents that exhibit an anti-herding behavior is included into the effective value of the social interaction, *J*′ = (1 − 2*f*_*c*_)*J*, where *f*_*c*_ is the fraction of contrarians. It is also evident from the results that even if some technologies attain an adoption level of almost 100%, others arrive to saturation with lower values of adoption. This is the main effect of contrarians. Maybe the word “contrarian” induces the idea of an irrational behavior, but the decision of non-adopting a given technology may have serious and rational basis, for example, in the case of the automobile, where the adoption is of the order of 90% in the USA, people who do not want to adopt the individual car may prefer public transportation, taxis, bikes or even walking. So, contrarians may have a relevant effect in the final adoption results, by avoiding saturation.

Moreover, the analysis of the data in three different periods, which represent changes of technological paradigms, according to long waves theory [15], contributes to reinforce the usefulness of the presented model, as well as to emphasize that the agent adoption velocity behaves differently, characterizing a faster spread today. It also agrees with the economic literature that studies the phenomenon of technological progress. In other words, by adding the periods of the three paradigms, we were able to show that the present model captures the particularity of the agent adoption of innovation speed in the different periods (paradigms) analyzed.

Another important point of the model is that a single curve fits all the innovations studied here, simply by changing the values of the pair (*A*, *J*′). Furthermore, we have shown that there is a clear correlation between the value of the parameters and the time of appearance of the innovation, according to the theory of economic waves. Today, social influence is probably the most relevant parameter, which can decides the future of the adoption of an innovation.

New technologies that have low “social penetration” may have difficulties to be adopted or maybe even not adopted. Some notable examples of products with penetration difficulties are smart glasses or robotic vacuum cleaners. Even large ad campaigns (*A*) do not guarantee adoption. As is clear from Fig 8 and Table 1, the values of advertising *A* and social interaction *J*′ are not independent.

The adoption of innovations could also be compared to the preference of opinions or even candidates for an election. However, the comparison is valid only up to a point. When it comes to elections, voters can generally choose between many candidates, whereas in our proposal there is only one option: to adopt or not a new technology, without branding options. We might think to applied our model to candidates for an election, associating the propaganda with term A and the candidate’s rejection of the resistance to adopt. However, even restricted to two candidates, the election process must be compared to choosing between two products, such as IOS and Android. This is a possible extension of the job.

The model may be improved, for instance by considering that both social influence or advertising, may change in time, and also the fact that some innovations maybe “overlapped by others”, like is the case of vinyl records, substituted by CD’s, also substituted by streaming services, and the VCR, gradually substituted by the DVD, itself substituted by the blue-ray, that was overlapped by streaming services. Another important point is to study adoption in other countries, particularly European or underdeveloped countries.

Our point here is to address successful innovations. However, most innovations, as well as ideas, and companies, are known to fail shortly after launch. In his classic book, Rogers, calls the early death of innovations, the adoption “chasm”. Other technologies are abandoned simply because new ones overcome them. One typical example is the VCR; you can see the plot for the VCR in Fig 1. Another interesting example is the Concorde aircraft, which survived for some time but was far from being fully adopted. Only two companies, British Airways and Air-France, had bought the planes and used them on relatively few routes. Some aspects of the dynamics of abandoning a technology were studied by some of us in Ref. [20].

Anyway, tackling the problem of failed technologies, or innovations with a very restricted diffusion process, must be done differently. Perhaps using an ecosystem model of innovations and their survival times or a mix of the present model with the model presented in “Dynamical model for competing opinions” (Ref. [27]). Other databases should also be used, with data on the consumption of different types of products and survival times, if available. We are currently working one these directions.

## Supporting information

S1 File (ZIP)
